# Efficacy of intermittent Theta Burst Stimulation (iTBS) and 10-Hz high-frequency repetitive transcranial magnetic stimulation (rTMS) in treatment-resistant unipolar depression: study protocol for a randomised controlled trial

**DOI:** 10.1186/s13063-016-1764-8

**Published:** 2017-01-13

**Authors:** Samuel Bulteau, Veronique Sébille, Guillemette Fayet, Veronique Thomas-Ollivier, Thibault Deschamps, Annabelle Bonnin-Rivalland, Edouard Laforgue, Anne Pichot, Pierre Valrivière, Elisabeth Auffray-Calvier, June Fortin, Yann Péréon, Jean-Marie Vanelle, Anne Sauvaget

**Affiliations:** 1 CHU de Nantes, Clinical Investigation Unit 18, Department of Addictology and Consultation-liaison Psychiatry, F-44000 Nantes, France; 2 University of Nantes, University of Tours, INSERM, SPHERE U1246, F-44000 Nantes, France; 3 CHU de Nantes, Department of Clinical Neurophysiology, F-44000 Nantes, France; 4 University of Nantes, Laboratory ‘Movement, Interactions, Performance’ (E.A. 4334), F-44000 Nantes, France; 5 CHU de Nantes, Department of Neuroradiology, F-44000 Nantes, France; 6 CHU de Nantes, Delegation of Clinical Research and Innovation, F-44000 Nantes, France

**Keywords:** Repetitive transcranial magnetic stimulation (rTMS), Intermittent Theta Burst Stimulation (iTBS), Treatment-resistant depression, Relapse

## Abstract

**Background:**

The treatment of depression remains a challenge since at least 40% of patients do not respond to initial antidepressant therapy and 20% present chronic symptoms (more than 2 years despite standard treatment administered correctly). Repetitive transcranial magnetic stimulation (rTMS) is an effective adjuvant therapy but still not ideal. Intermittent Theta Burst Stimulation (iTBS), which has only been used recently in clinical practice, could have a faster and more intense effect compared to conventional protocols, including 10-Hz high-frequency rTMS (HF-rTMS). However, no controlled study has so far highlighted the superiority of iTBS in resistant unipolar depression.

**Methods/design:**

This paper focuses on the design of a randomised, controlled, double-blind, single-centre study with two parallel arms, carried out in France, in an attempt to assess the efficacy of an iTBS protocol versus a standard HF- rTMS protocol. Sixty patients aged between 18 and 75 years of age will be enrolled. They must be diagnosed with major depressive disorder persisting despite treatment with two antidepressants at an effective dose over a period of 6 weeks during the current episode. The study will consist of two phases: a treatment phase comprising 20 sessions of rTMS to the left dorsolateral prefrontal cortex, localised via a neuronavigation system and a 6-month longitudinal follow-up. The primary endpoint will be the number of responders per group, defined by a decrease of at least 50% in the initial score on the Montgomery and Asberg Rating Scale (MADRS) at the end of rTMS sessions. The secondary endpoints will be: response rate 1 month after rTMS sessions; number of remissions defined by a MADRS score of <8 at the endpoint and 1 month after; the number of responses and remissions maintained over the next 6 months; quality of life; and the presence of predictive markers of the therapeutic response: clinical (dimensional scales), neuropsychological (evaluation of cognitive functions), motor (objective motor testing) and neurophysiological (cortical excitability measurements).

**Discussion:**

The purpose of our study is to check the assumption of iTBS superiority in the management of unipolar depression and we will discuss its effect over time. In case of a significant increase in the number of therapeutic responses with a prolonged effect, the iTBS protocol could be considered a first-line protocol in resistant unipolar depression.

**Trial registration:**

ClinicalTrials.gov, Identifier NCT02376491. Registered on 17 February 2015 at http://clinicaltrials.gov.

**Electronic supplementary material:**

The online version of this article (doi:10.1186/s13063-016-1764-8) contains supplementary material, which is available to authorized users.

## Background

Depression is a real public health problem. In 2020, according to the World Health Organisation (WHO), depression will be the second major cause of handicap and premature death in the world after coronary diseases. Standard treatment comprises support therapy combined with medication. However, at least 40% of patients do not respond to the initial treatment and 20% present with persistent resistance to conventional pharmacological treatments [1]. It is, therefore, essential to find efficient treatment alternatives for resistant depression. The reference treatment in this case is still electroconvulsive therapy (ECT) with a 48% response rate in the event of severe resistance to pharmacological therapies [2]. Repetitive transcranial magnetic stimulation (rTMS) is a non-invasive, focal, cortical stimulation technique involving modulation of cortical excitability. It is an upstream alternative to ECT and could close to match comparable efficacy in the absence of psychotic symptoms with a satisfactory duration and number of stimuli [3, 4]. Its interest in the treatment of resistant unipolar depression has clearly been established in conjunction with antidepressant chemotherapy.

The efficacy of rTMS depends on its parameters (stimulation site, orientation of the magnetic field, number of stimuli delivered as well as the frequency, intensity and duration of stimulation) [5]. The two types of dorsolateral prefrontal cortex (DLPFC) stimulation used in the treatment of depression – high frequency to the left (L-HF) and low frequency to the right (R-LF) – have proved to be equi-effective [6–9]. Response rates on average vary from 30 to 40% depending on the series [10, 11], with a 58% response rate maintained at 3 months, then 33% at 6 months [12, 13].

Although these various parameters currently have yet to be optimised, a new rTMS technique known as Theta Burst Stimulation (TBS) has recently emerged. This displays faster, more robust action compared to conventional protocols [14], with excellent tolerability [15], provided that safety recommendations are followed [16]. Two different methods have been described: intermittent (iTBS) and continuous Theta Burst Stimulation (cTBS) with facilitating and inhibitory effects, respectively. iTBS involves the application of bursts of three pulses at a frequency of 50 Hz every 200 ms; therefore, at 5 Hz, delivery is over 2 s and repeated every 10 s, 20 times in succession. An uncontrolled study on a small sample generated 70% of responses and 42% of remissions in resistant unipolar depression following a course of iTBS [17]. These shorter sessions would be an additional source of comfort for the patient not to mention the lower cost of the session. It should be noted that a very recent study highlighted the superiority of iTBS over cTBS and placebo in the management of resistant depression [18]. In 2014, Bakker et al. demonstrated similar efficacy with iTBS prefrontal dorsomedial bilateral stimulation (6 min) and a 10-Hz protocol (30 min) in an uncontrolled retrospective study [19]. With regard to recent scientific literature, various points must be specifically investigated: controlled comparison of protocols with the enrolment of a homogeneous population in terms of treatment and clinical form of the condition (most studies combine both bipolar and unipolar disorder); duration of the effect with longitudinal follow-up; and predictive response factors [20]. The main predictive therapeutic response factors identified to date are age, duration of the episode, degree of therapeutic resistance, clinical profile and, in particular, cognitive impairment and psychomotor retardation [21, 22]. A study of cortical excitability, which reflects the gabaergic and glutamatergic transmission of cortical interneurones (IN), could also prove promising in distinguishing neurostimulation responder profiles. No controlled study comparing the efficacy of iTBS versus conventional rTMS protocols in unipolar resistant depression has been published to date.

### Specific aims

The aim of this randomised controlled trial is to investigate the efficacy of iTBS versus 10-Hz high-frequency transcranial magnetic stimulation (HF-rTMS) in a population of patients suffering from unipolar depression (Montgomery and Asberg Rating Scale (MADRS) score of >20) despite the use of two antidepressant agents during the current episode. Moreover, this trial will evaluate whether certain specific clinical or neuropsychological dimensions, objective measurements of psychomotor retardation or even distinct cortical excitability profiles could predict the efficacy of iTBS and HF-rTMS, respectively.

## Methods/design

### Study design, setting and recruitment

**Fig. 1:**
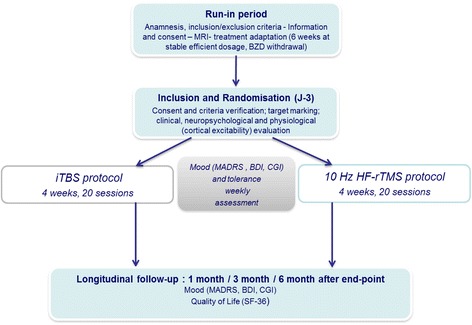
Flow Chart

This is a randomised, controlled, double-blind, single-centre study. It is carried out at the Centre Hospitalier Universitaire (University Hospital Centre) of Nantes, France. The patients are randomly assigned to the iTBS or the HF-rTMS group as shown in Fig. 1 (Flow Chart). More specifically, the two parallel arms compare an iTBS protocol to a so-called conventional protocol at 10 Hz [23]. The stimulation target is identical in the left dorsolateral prefrontal cortex (Brodmann areas 9 and 46), localised via Nextim® (software Eximia®) neuronavigation. Participants are randomised to one of the treatment groups using a computer programme included in the electronic Case Report Form (e-CRF). This study has been approved by the local Nantes Ouest IV Ethics Committee (reference: ID RCB N°2014-AO1918-39) and compiled in accordance with the principles of the Declaration of Helsinki (final version 2004) as well as French legislation (article L1121-160 and L1126-7 of the Public Health Code). Additional quality standards are detailed in Additional file 1 according to SPIRIT Check-list requirements. All of the patients are given written and verbal information about the study aim and procedures. They sign a written Consent Form in order to take part in the study. The aim is to enrol 60 patients who have been put forward either by private or hospital psychiatrists in the region, previously informed in writing of the study.

### Inclusion criteria

The 60 patients between 18 and 75 years old must present with a current depressive episode considered major (defined by *Diagnostic and Statistical Manual of Mental Disorders*, *fifth edition* (DSM-V) diagnostic criteria [24] and a MADRS score of >20 [25]) and resistant (failure to respond to two sequences of different antidepressants at an effective dose level over a period of 6 weeks during the current episode). The current antidepressant is continued at a stable dose throughout the study.

Each subject must be able to: understand the information; take a decision; volunteer to participate; complete the required questionnaires; take orally administered treatment independently or have the necessary assistance to do so throughout the study; and return to the research centre for successive visits.

### Noninclusion criteria

Patients presenting with at least one of the following criteria are not be enrolled in the study: diagnosis of a bipolar disorder; schizophrenia; addiction; neurodegenerative disease; use of benzodiazepines (unless prescribed over 3 months earlier at a stable dosage); use of mood-modifying treatments (thyroid extracts, interferon, corticosteroids); previous failure of ECT therapy; anticonvulsant treatment; contraindication to magnetic resonance imaging (MRI); contraindication to the practice of rTMS: history of convulsions, progressive neurological and neurosurgical disorders; any prosthetic material or foreign body in situ (pacemaker, implantable defibrillator); minors or persons deprived of liberty following a legal or administrative decision or hospitalised without consent, in guardianship; or pregnant women or women of child-bearing age who are not using contraception because of no available data about iTBS and pregnancy (these women could receive, if absolutely necessary, from case to case, conventional rTMS treatment, outside the study). The same applies for subjects unable to guarantee longitudinal follow-up.

In case of a serious adverse event or exacerbated symptoms of depression, blind status will be lifted and patients will receive appropriate care and retained in the follow-up.

### Study process

The screening visit V1 (D − 21 to D42) includes the Patient Information Leaflet, collection of the Consent Form and checking of inclusion/exclusion criteria. The medical research team will be in charge of enrolment and assignation of participants to the intervention. The study manager and psychiatrist investigators can generate the allocation sequence. Treatment is adjusted in line with the course of therapy. A clinical examination is also carried out and MRI prescribed to rule out any neurological disorder and mark the neuronavigation target during the baseline visit. The procedure is conducted using a Siemens 1.5 T machine with the following sequences: diffusion, T1 3D, FLAIR 2D and T2 rapid and SWI.

#### Randomisation

During the baseline inclusion visit, the inclusion/exclusion criteria are checked and participants randomised into two groups by a computerised random number generator with a permuted block design (ratio 1:1) without stratification or minimisation. Block size and type of variation (fixed or randomly) are not yet known by the investigators to maintain adequate blinding. Randomisation occurs in a recorded delay of maximum 72 h before first rTMS session.

#### Blinding

Research nurses, who perform rTMS sessions, are the only persons to know the allocated sequence. Just after the randomisation, they will receive an automatic e-mail on their individual professional mail box. The session will take place in a dedicated room with appropriate noise control and a signal on the door during the session to prevent accidental entry of investigators. Patients are not told the group they belong to, will not have precise description of rTMS parameters and duration and will not be able to speak to each other. Both treatments will be equally presented as efficient and superior to placebo.

#### Follow-up

Investigators will meet participants 1, 3 and 6 months after the last rTMS session.

A patient who fails to respond to therapy (no decrease of over 50% in the MADRS score) despite 4 weeks of treatment will continue longitudinal follow-up in order to avoid selection bias. A telephone call is made in the second, fourth and fifth month outside the follow-up consultations to keep in contact with patients and remind them of the next appointment in order to avoid their being lost to follow-up.

The study will last 33 months in total with 24 months being the enrolment period. Patients are followed up over a period of approximately 34 weeks in total (Table 1).

**Table 1:** Flow chart

Clinical trial										
Study period	Pre-enrolment	Inclusion and randomisation	Trial period							
			rTMS treatment					Follow-up phase		
Clinical visits	V1 information	V2 (D − 3)	V3 AD0 Start of rTMS treatment	V4 D7 (±2D) After 5 sessions	V5 D14 (±2D) After 10 sessions	V6 D21 (±2D) After 15 sessions	V7 D28 (±2D) After 20 sessions	V8 M1 (±7D)	V9 M3 (±7D)	V10 M6 (±7D)
Length of time compared to randomisation	−21 to 42 days	0								
Inclusion/exclusion criteria	X	X	X	X	X	X				
Signed Consent Form	X	Verification								
Medical history	X									
MRI	X	Target labelling								
Concomitant treatments	X	X	X	X	X	X	X	X	X	X
Motor threshold titration			X	X	X	X	X			
Routine clinical examination	X	X	X	X	X	X	X	X	X	X
MADRS, CGI, BDI		X		X	X	X	X	X	X	X
SAS, SHAPS, MoCA, ERD, HARD		X					X	X		
MSM, TCI		X								
TMT MCST Verbal fluency		X					X	X		
FTT Prehensile motor strength Reaction times		X					X	X		
SF-36		X					X	X	X	X
Adverse events			X	X	X	X	X	X	X	X
Ancillary cortical excitability study										
Biophysical measurements			X				X			
Signed Consent Form	X	Verification								

*BDI* Beck Depression Inventory, *CGI* Clinical Global Impression, *ERD* Echelle de Ralentissement Dépressif, *FTT* Finger Taping Test, *HARD scale* Humeur, Anxiété, Ralentissement, Danger, *MADRS* Montgomery and Adsberg Rating Scale, *MCST* Modified Card Sorting Test, *MoCA* Montreal Cognitive Assessment, *MRI* magnetic resonance imaging, *MSM* Maudsley Staging Model, *rTMS* repetitive transcranial magnetic stimulation, *SAS* Starkstein’s Apathy Scale, *SF-36* Short-Form 36, *SHAPS* Snaith-Hamilton Pleasure Scale, *TCI* Temperament and Character Inventory, *TMT* Trail Making Test

### Assessments

The following variables are documented during the baseline visit: sociodemographic (age, gender, laterality, professional and marital status); medical history (length of illness, duration of current episode; psychiatric and addictive comorbidities; somatic history; treatments prescribed; degree of prior therapeutic resistance according to the Maudsley Staging Model (MSM) [29]; basal personality according to Cloninger’s Temperament and Character Inventory (TCI) [30]).

The following variables are evaluated at the beginning and end of treatment, as shown in Table 1: intensity of the depression according to the MADRS [25]; the Beck Depression Inventory 13 items (BDI 13) [26] and Clinical Global Impression – Severity (CGI-S) [27]; and quality of life with the Short-Form 36 Health Survey (SF-36) [28]. Response will be defined by a 50% reduction of MADRS or BDI score, and remission by a MADRS score of <8 and a BDI score of <10.

Potential explanatory variables to predict treatment response in addition to sociodemographic and anamnestic data are: clinical dimensions according to the Echelle de Ralentissement Dépressif (ERD) (Depression Retardation Scale) [31], the lack of pleasure Snaith-Hamilton Pleasure Scale (SHAPS) [32] and Starkstein’s Apathy Scale (SAS) [33]; cognitive functions according to the Montreal Cognitive Assessment (MoCA) [34], the Verbal Fluency Test [35] [36], the Modified Card Sorting Test (MCST) [37], the digit span, the Wechsler Adult Intelligence Scale (WAIS IV) [38] and the Trail Making Test [39]; psychomotor retardation using specific tests such as the Finger Taping Test (FTT) [40, 41], the prehensile motor strength test [42], a test to measure information processing speed (currently being validated by our team [43]); and neurophysiological measurements of cortical excitability, namely the cortical motor threshold, short intracortical inhibition (SICI) and intracortical facilitation (ICF) expressed as a percentage compared to the base value, and similarly for the cortical silent period (CSP), which is carried out in both hemispheres [44–46]. All these scales are validated scales. Concerning the motor retardation assessment, we recently showed that administering a battery of psychomotor tests during rTMS sessions is feasible, free of adverse effects and well-tolerated by that population [43]. Cortical excitability is used in daily practice for patients with neurodegenerative troubles with an excellent tolerance and feasibility. Those measures are not performed the same day (interval maximum 72 h) to avoid participant overstimulation. An incentive of €80 is planned for cortical excitability ancillatory measures.

The following scales are evaluated on a weekly basis: the MADRS, the BDI and the CGI to investigate the response kinetics and 1 month, 3 months and 6 months after the endpoint as well as quality of life (SF-36). Data collected directly from participants themselves will be the BDI, the SAS, the SHAPS, the TCI and the SF-36. Adherence to study quality standards is carried out by an independent research associate. All significant adverse events will be reported in the e-CRF by the research team.

### Interventions

For the rTMS, an eight-shaped coil (Cool B65) and a Magpro Stimulator X100 (Dantec Company, Copenhagen, Denmark) are used. The resting motor threshold (RMT) is recorded daily by a Natus Keypoint® (Natus, Middleton, WI, USA). This is defined as the intensity required to elicit at least five motor-evoked potentials (MEPs) with a 50-μV peak-to-peak amplitude out of ten consecutive stimulations when the coil is placed over the left primary cortex (site for maximal stimulation of the abductor pollicis brevis muscle). The parameters used for HF-rTMS delivery are: 110% of RMT; 10 Hz; 20 min; 4 s per train; 28 s intertrain interval; 1600 pulses per day (40 trains of 40 pulses each). The iTBS protocol will be: 80% of RMT; 50 Hz; 6 min; 600 pulses a day.

The target is left DLPFC corresponding to the junction between Brodmann areas 9 and 46 according to an individual’s 3D-MRI. During rTMS sessions, participants are instructed to keep their eyes open and to be relaxed. All subjects are evaluated before, once a week during the rTMS course, at the endpoint and then at 1, 3 and 6 months.

### Primary outcome measures

The primary endpoint in our study will be the number of responders per group, defined by a decrease of at least 50% in the initial MADRS score at the end of rTMS sessions.

### Secondary outcome measures

The secondary endpoints will be: therapeutic response rate corresponding to a MADRS score improvement of >50% in each group 1 month after rTMS sessions; number of remissions defined by a MADRS score of <8 at the endpoint and 1 month after; number of therapeutic responses and remissions maintained in the 6 months following rTMS treatment; changes in quality of life; and clinical, motor, neuropsychological and neurophysiological (cortical excitability) therapeutic response markers.

### Statistical analysis

A descriptive analysis of the data collected during each patient evaluation will be carried out up until the final evaluation. Specific time points for analysis are: end of rTMS sessions and, 1, 3 and 6 months after the treatment course. Continuous variables will be described using median and range; and qualitative variables using frequencies and percentages.

The final analysis will be conducted according to the intent-to-treat (ITT) principle. The starting hypothesis is a 25% response rate in the 10-Hz group in accordance with 10-Hz HF-rTMS trials of reference [5] and 60% in the iTBS group based on pilot studies [17]. Assuming a 5% (bilateral) type I error and a power of 80%, a total of 60 subjects is required (calculated using SAS software).

The therapeutic response rates (proportion of responders) in each group will be compared using a chi-squared test (or Fisher’s exact test, if appropriate). Estimates of absolute and relative differences (via the odds ratio or relative risk) in terms of efficacy will be provided with their corresponding 95% confidence intervals. The format of the outcome data used for each participant for analysis will be changes from baseline.

Changes in quantitative variables over time (evaluation of the therapeutic effect of rTMS (iTBS) on variations in the MADRS, BDI and CGI scores) will be analysed using random effect models allowing to take into account the repeated measurements. Time (baseline, after 5, 10, 15, and 20 sessions, +1 month, +3 months and +6 months) and group (iTBS or standard) effects will be estimated and tested as well as an interaction with group. Changes in other quantitative variables over time will be investigated according to the same strategy.

The analysis and comparison between groups of the onset of relapse of depression in the 6 months will be carried out using a logistic regression model and testing factors linked to the therapeutic response (neuropsychological and motor tests, dimensional scales, initial measurements of cortical excitability).

Concerning the risk of missing data, a very low attrition rate is expected, below 5% [47] because of the well-known high tolerance to rTMS and iTBS [48]. In case of withdrawal of consent, patients will not be included in final analysis – they will be replaced.

Missing data will be described in terms of frequencies and percentage for each group. Imbalances will be evaluated by the chi-squared test (or Fisher’s exact test). Comparison of missing data onset during follow-up will be realised with a log-rank test for longitudinal data. Each dropout will be described as follows: arm, exit date, exit reason, characteristics at inclusion and last data collected. In case of missing data, despite every effort to prevent it, a multiple imputation analysis will be performed.

## Discussion

Depression is known as a difficult-to-treat disorder. By 2013, unipolar depression is expected to rank second out of the 15 most common disorders after AIDS and before ischemic heart disease [49]. The advantages of rTMS are numerous: it is painless, well-tolerated in terms of memory, cardiac, hepatic and renal functions as well as libido. There is no need for general anaesthesia with curarisation. Since the early 1990s, an increasing number of studies has focused on the therapeutic potential of rTMS in psychiatry [50].

This technique has a compliance rate of 97% (versus 60% traditionally recorded for medication). In fact, several meta-analyses based on randomised, controlled, double-blind studies refer to the therapeutic efficacy of rTMS in the management of resistant depression. The use of rTMS devices was also validated for this indication by the US Food and Drug Administration (FDA) in 2008 and by the European Union (EU) in 2012. By way of example, one of the most recent analyses highlighted a three-fold greater response and remission rate for left prefrontal cortex stimulation at high frequency versus placebo [10].

Furthermore, once antidepressants have failed, rTMS would be less expensive and would allow a better quality of life and greater function compared to conventional treatment strategies [51]. Its mechanisms of action are manifold: improvement in prefrontal hypometabolism [52] and neuromodulation of remote cerebral areas (especially the subgenual region) [53], regulation of the hypothalamo-hypophyseal axis [53], modulation of cortical excitability and synaptic plasticity [54–56] and dopaminergic secretion [57, 58].

The optimisation of alternative treatments for refractory or chronic depression is, therefore, a public health issue. The Theta Burst paradigm seems promising since 3 min of iTBS are essentially more effective and have a longer lasting effect than 20 min of conventional rTMS stimulation at 5 Hz (as evidenced in MEPs) [59]. In fact, iTBS tends to mimic the physiological rhythm of the human neocortex and involves the cerebral plasticity mechanisms responsible for positive and presumably longer-lasting effects [60], with a shorter, less intense stimulation mechanism [61]. Duprat et al., (2016) recently found a 30% remission rate (HDRS <7) after 2 weeks of accelerated (20 sessions/2 weeks) iTBS in a cross-over, sham-controlled design [48]. These facts suggest additional patient comfort, lower session costs and greater prevention of relapse. In the case of a prolonged effect, it could be an alternative to potentiating medicinal treatment (combinations of antidepressant, mood stabilising or adjuvant antipsychotic agents) with fewer compliance problems. The efficacy of Theta Burst was investigated in the management of resistant depression in two studies, which confirmed its superiority compared to placebo (Li et al., [18] with 30 subjects; Plewnia et al., [62] involving 60 subjects). Recently, in a larger sample but under natural conditions (retrospective study), iTBS was as clinically effective as HF-rTMS at 10 Hz but five times longer (6 versus 30 min, respectively) [19]. The authors concluded that these data should be confirmed by randomised controlled trials. Better targeting of the responder profile leads to better use of human and material resources in medical-economic terms. In cognitive terms, the planning assessed in the TMT, mental flexibility assessed using the MCST, or verbal fluency are influenced by rTMS treatment [63–67] and are, therefore, potential response markers. Psychomotor retardation has a predictive value in assessing the response for most biological treatments of depression [68]. Unlike cognitive functions, its pure motor component has not been assessed objectively to date in the context of the cerebral stimulation techniques used in the management of depression, for instance, with tasks such as the FTT or the prehensile motor strength test. The three main parameters affecting the motor cortex excitability are the CSP partly reflecting the activity of the gamma-aminobutyric acid (GABA)-B inhibitory circuits and double-pulse measurements including: SICI partly reflecting the activity of GABA-A IN inhibitors; and ICF partly reflecting the activity of glutamatergic IN activators. Two recent meta-analyses have highlighted changes in motor cortical excitability in an entire series of psychiatric disorders compared to healthy subjects [69, 70], with essentially an alteration in SICI and CSP during the depressive episode. In recent years, a few authors have focused on their potential as response markers, especially during noninvasive brain stimulation. In 2008, for instance, Lefaucheur et al., highlighted interhemispheric asymmetry of cortical excitability in depressed subjects: CSP and SICI were altered in the left hemisphere compared to the right and contrary to findings in healthy subjects [71]. Following cerebral stimulation (ECT and rTMS), Bajbouj et al. showed that CSP and SICI improved in responders and not in nonresponders [72].

To date and to our knowledge, this is the first trial aiming to investigate the comparative influence of iTBS and HF-rTMS on psychomotor retardation, executive functions and cortical motor excitability in depressed subjects. Psychomotor retardation could be corrected by the pro-dopaminergic effect of rTMS. Furthermore, executive functions may be a marker of the effect of DLPFC stimulation [73]. Given the data available on the effect of rTMS on magnetic resonance spectroscopy measurements, which indicate an increase in GABA and glutamate levels after rTMS [74, 75], neuromodulation can be assumed to manifest in the form of changes in cortical excitability measurements tending towards normalisation with 10-Hz protocols and iTBS, the greatest change being apparent in the case of the latter. The intensity of the gabaergic and/or glutamatergic deficit(s) and the degree of interhemispherical asymmetry in cortical excitability tests could identify responder patient profiles for both treatments. Moreover, measuring the difference in neurophysiological effect in both protocols would enhance the neurobiological understanding of this disease.

This sample is probably not entirely representative of a population often combining comorbidities with considerable treatment heterogeneity. However, we opted to limit the inclusion criteria in order to facilitate comparison with fewer confusing variables and interpret cortical excitability measurements.

### Trial status

Patient recruitment is still ongoing.

## Additional file

Additional file 1: SPIRIT 2013 Checklist. recommended items to address in a clinical trial protocol and related documents. (DOCX 52 kb)

## Abbreviations

BDI: Beck Depression Inventory
CGI: Clinical Global Impression
CSP: Cortical Silent Period
cTBS: Continuous Theta Burst Stimulation
DLPFC: Dorsolateral Prefrontal Cortex
e-CRF: Electronic Case Report Form
ECT: ElectroConvulsive Therapy
ERD: Echelle de Ralentissement Dépressif
EU: European Union
FDA: Food and Drug Administration
FTT: Finger Tapping Test
GABA: Gamma-AminoButyric Acid
HARD scale: Humeur, Anxiété, Ralentissement, Danger
ICF: Intracortical Facilitation
IN: InterNeurons
iTBS: Intermittent Theta Burst Stimulation
ITT: Intention-To-Treat
L-HF: Left-High Frequency
MADRS: Montgomery and Adsberg Rating Scale
MCST: Modified Card Sorting Test
MEP: Motor-Evoked Potential
MoCA: Montreal Cognitive Assessment
MRI: Magnetic Resonance Imaging
MSM: Maudsley Staging Model
R-LF: Right-Low Frequency
RMT: Resting Motor Threshold
rTMS: repeated Transcranial Magnetic Stimulation
SAS: Starkstein’s Apathy Scale
SF-36: Short-Form 36
SHAPS: Snaith-Hamilton Pleasure Scale
SICI: Short Intracortical Inhibition
TCI: Temperament and Character Inventory
TMT: Trail Making Test
WAIS IV: Wechsler Adult Intelligence Scale
WHO: World Health Organisation
