# Supplementary material for: Efficacy of intermittent Theta Burst Stimulation (iTBS) and 10-Hz high-frequency repetitive transcranial magnetic stimulation (rTMS) in treatment-resistant unipolar depression: study protocol for a randomised controlled trial
Source: Trials. 2017 Jan 13;18:17. doi: 10.1186/s13063-016-1764-8 (PMC5237321; doi:10.1186/s13063-016-1764-8)
Supplement: Additional file 1: — SPIRIT 2013 Checklist. recommended items to address in a clinical trial protocol and related documents. (DOCX 52 kb) [file 13063_2016_1764_MOESM1_ESM.docx]

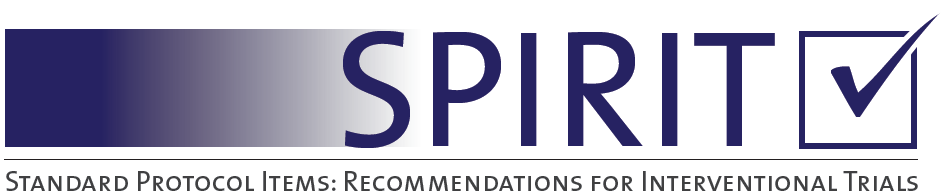


SPIRIT 2013 Checklist: Recommended items to address in a clinical trial protocol and related documents*

| Section/item | ItemNo | Description |
| --- | --- | --- |
| **Administrative information** | | |
| Title | 1 | Efficacy of intermittent Theta Burst Stimulation (iTBS) and 10 Hz High frequency rTMS in treatment-resistant unipolar depression: study protocol for a randomized controlled trial. |
| Trial registration | 2 | <http://clinicaltrials.gov/>: NCT02376491 |
| Protocol version | 3 | 15/06/2014. AOI2014 |
| Funding | 4 | Internal call for bids CHU Nantes |
| Roles and responsibilities | 5a | Samuel Bulteau conceived the study and designed the study protocol and is the coordinator of the study. Samuel Bulteau, Annabelle Bonnin, Anne Sauvaget wrote the manuscript. Samuel Bulteau, Anne Sauvaget, Edouard Laforgue, Jean-Marie Vanelle are investigators. June Fortin sought ethical and regulatory approval and contribute to global organization and coordination. Annabelle Bonnin is responsible for e-CRF completion. Anne Pichot and Pierre Valrivière contribute to the technique and treatment planification. Veronique Sébille is responsible for statistical power calculation and analysis of data as well as writing of the manuscript for these parts. Thibaut Deschamps and Veronique Thomas-Ollivier are responsible for designing and assessing neuropsychological status and psychomotor retardation. Elisabeth Auffray-Calvier is responsible for MRI data. Samuel Bulteau, Guillemette Fayet and Yann Péréon are responsible for cortical excitability assessment. |
|  | 5b | June Fortin, project manager  CHU de Nantes, Delegation of Clinical Research and Innovation |
|  | 5c | Study sponsor and funder is CHU de Nantes. Study design, analysis, and interpretation of data as well as writing of the report and the decision to submit the report for publication is under authors responsibility. Collection and management quality will be ensure by an independent research associate. |
|  | 5d |  |
| Introduction |  |  |
| Background and rationale | 6a | New rTMS protocol, named TBS (Huang et al 2005) shows some promise (Holzer et al., 2010, Li et al., 2014, Bakker et al., 2014) and are available but to date no comparative trial with standard high frequency TMS in depression has been published.  This study take into account some gaps in literature in that field (Dell'Osso et al., 2011): protocol controlled comparison, search of response markers (clinical dimensions, neuropsychological and physiological), adequate TMS duration and treatment association, use of neuronavigation, longitudinal follow-up. |
|  | 6b | 10 Hz high frequency rTMS on the left dorsolateral prefrontal cortex is to date the most used TMS protocol for depression worldwide and its use was validated by FDA in 2008 (O'Reardon et al., 2007) |
| Objectives | 7 | The main hypothesis is that there will be a better response rate (defined as a reduction of 50% of MADRS score) in iTBS group versus 10 Hz HF-rTMS group. |
| Trial design | 8 | It is a superioty randomized controlled trial with two parallel groups and a 1:1 allocation ratio |
| Methods: Participants, interventions, and outcomes | | |
| Study setting | 9 | The study takes place in a single centre: Nantes University Hospital |
| Eligibility criteria | 10 | *Inclusion criteria*  -18-75 years old  -current depressive episode considered major (defined by DSM-V diagnostic criteria and a Montgomery Asberg Depression Rating Scale (MADRS) score of > 20  -resistant (2 sequences of different antidepressants at an effective dose level over a period of 6 weeks during the current episode).  -Each subject must be able to: understand information; take a decision; volunteer to participate; complete the required questionnaires; take oral treatment independently or have the necessary assistance to do so throughout the study; and return to the research centre for successive visits.  *Non-inclusion criteria*  diagnosis of a bipolar disorder (subtype 1 or 2); schizophrenia; addiction; neurodegenerative disease; use of benzodiazepines (unless prescribed over 3 months earlier at a stable dosage); use of mood-modifying treatments; previous failure of ECT therapy; anticonvulsant treatment; contraindication to Magnetic Resonance Imaging (MRI); contraindication to the practice of rTMS: history of convulsions, progressive neurological and neurosurgical disorders, any prosthetic material or foreign body (pacemaker, implantable defibrillator); minors or persons deprived of liberty following a legal or administrative decision or hospitalised without consent, in guardianship; or pregnant women. |
| Interventions | 11a | 20 sessions, delivered by a eight-shaped coil (Cool B65) and a Magpro Stimulator X100, of either iTBS (80% of RMT; 50 Hz; 6 minutes; 600 pulses a day) or 10 Hz-TMS(110% of RMT; 10 Hz; 20 minutes; 4 seconds per train; 28 seconds inter-train interval; 1600 pulses per day, 40 trains of 40 pulses each) on the DLPFC under neuronavigation. |
|  | 11b | Criteria for discontinuing: drug dose change, participant request, significant worsening of the disease |
|  | 11c | Strategies to improve adherence to intervention protocols: good selection and information. 80 euros for cortical excitability ancillatory assessment. Phone call between visits in longitudinal follow-up. |
|  | 11d | Concomitant support psychotherapy is tolerated but no structured intervention as CBT. No treatment modification is allowed during treatment course. After end point in case of relapse in longitudinal follow-up, treatment modifications are allowed but notified in e-CRF |
| Outcomes | 12 | Primary outcome: proportion of responders (50% improvement compared to baseline) according to MADRS score in each group at the end of treatment course. Secondary outcomes: proportion of responders one month after TMS sessions; number of remissions (defined by MADRS score < 8) at end point and one month after; number of therapeutic responses and remissions maintained in the 6 months following rTMS treatment; changes in quality of life; clinical, motor, neuropsychological, and neurophysiological (cortical excitability) therapeutic response markers. |
| Participant timeline | 13 | Please see Table 1 and Figure 1. |
| Sample size | 14 | The starting hypothesis is a 25% response rate in the 10-Hz group in accordance with 10Hz HF-rTMS trials of reference [5] and 60% in the iTBS group based on pilot studies [17]. Assuming a 5% (two-sided) type I error and a power of 80%, a total of 60 subjects is required |
| Recruitment | 15 | Information given to private practitioners and in local media. Research staff weekly to screen patients seen in consultation or hospitalisation. |
| **Methods: Assignment of interventions (for controlled trials)** | | |
| Allocation: |  |  |
| Sequence generation | 16a | Computer random number generator with a permuted block design (ratio 1:1) without stratification nor minimisation. Block size and type of variation (fixed or randomly) are not yet known by investigators but can be provided separately if required. |
| Allocation concealment mechanism | 16b | Allocation sequence is automatically generated and provided to research nurse on their personal professional mail. |
| Implementation | 16c | Generation of the allocation sequence: research associate or investigators. Participants enrolment: psychiatrists investigators. Assignation of participants to interventions: computerised randomisation |
| Blinding (masking) | 17a | Outcome assessors, data analysts and patients will be completely blinded with no mean to know the protocol (restricted access to treatment room during session, automatic allocation). Patients will not be told the allocation sequence. Both treatments are equally presented as efficient and superior to placebo. No information is given concerning precise differences between both protocol in terms of parameters and duration. Care providers (nurse delivering TMS session) will know allocation sequence but will not be involved in assessment and are trained and familiar with clinical therapeutic trials. |
|  | 17b | Unblinding is permissible in case of significant worsening of the episode during TMS course calling for an adaptation of therapeutic strategy or in case of serious adverse event. Data manager will inform which can in turn inform the patient. |
| **Methods: Data collection, management, and analysis** | | |
| Data collection methods | 18a | Plans for assessment and collection of data is precised in Table 1. Assessors are trained psychiatrists with a daily experience of heteroquestionnaire in mood disorders. A same patient will be assessed by the same investigator during the protocol, or replaced in exceptionally circumstances. Research associate will ensure that there is no missing data throughout e-CRF completion with a real time monitoring. All questionnaire are validated and widely used in literature in the field (cf reference list). Cortical excitability measurements are under the responsibility of trained neurophysiologists of a reference centre for muscular and neurodegenerative diseases. Psychomotor battery was established by a dedicated specialised team and whose work was subject to publication. All data collection forms are provided in annex in the protocol |
|  | 18b | Participant retention will be ensure by adequate selection (disponibility, motivation, complete information, adequate planification...), an incentive of 80 Euros is planned for cortical excitability ancillatory tests, and a telephone call is made in the second, fourth and fifth month outside the follow-up consultations to keep in contact with patients and remind them of the next appointment  In case of discontinuation or deviation of intervention protocols patients will be invited to complete longitudinal follow-up (mood quality of life), and the reasons of this discontinuations/deviations registered as well as all medication changes. |
| Data management | 19 | Data will be entered anonymously (only the initials and inclusion number will be reported) by research associate or investigators in a secured e-CRF (with confidential password). The e-CRF generate some queries if data are missing. Data management procedure can be found in the protocol and also precised by data-managers from Delegation of Clinical Research and Innovation of CHU de Nantes |
| Statistical methods | 20a | Descriptive analysis (median, range, percentage)  intention to treat principles for final analysis with 5% two-sided type 1 error risk and power of 80%  comparison of responders proportion with Chi-2 test (or Fisher's test if appropriate)  95 % confidence interval for absolute and relative differences (via the Odds ratio or relative risk) in terms of efficacy  changes in quantitative variables over time will be analysed with random effect models.  Time, and group effects will be tested along with their interaction  Onset of relapse in both groups will be tested with logistic regression model and testing potential response factors. |
|  | 20b |  |
|  | 20c | Missing data will be described in terms of frequencies and percentage for each group. Imbalances will be evaluated by Chi-2 test (or Fisher's exact test). Comparison of missing data onset during follow-up will be realized with a log-rank test for censored data. Each dropout will be described according to treatment arm, exit date, exit reason, characteristics at inclusion and last data collected. Multiple imputations will be performed in case of missing data |
| **Methods: Monitoring** | | |
| Data monitoring | 21a | An Internal independent data monitoring is planned. One visit of a research associate not involved in the study has already occurred. |
|  | 21b | No interim analyses is envisaged. |
| Harms | 22 | All adverse effect are reported in the e-CRF. Specific validated forms to declare serious adverse event are available in the protocol. |
| Auditing | 23 | An annual audit by the Delegation of Clinical Research and Innovation of CHU de Nantes is provided. |
| Ethics and dissemination | | |
| Research ethics approval | 24 | ANSM approval (registration number DMDPT-BLOC/MM/2014-AO1918-39/MS 1)  Nantes Ouest IV Ethics Committee approval 1^st^ december 2015 (reference 05/15, TLT/BB CPP N°738/2015) |
| Protocol amendments | 25 | No protocol amendments is needed. |
| Consent or assent | 26a | Investigating physicians will obtain informed consent or assent from potential trial participants or authorised surrogates, thanks to an approved paper consent form with a carbon copy for the patient. |
|  | 26b | NA |
| Confidentiality | 27 | Personal information about potential and enrolled participants will be either stored in the medical record or in research record and identified with initials and number of inclusion. Records are stored securely in locked areas. |
| Declaration of interests | 28 | Authors have no conflicts of interest to declare. |
| Access to data | 29 | Data-manager, study statistician, principle investigator, and the two person in charge of cortical excitability and psychomotor assessment will have access to the final trial dataset. |
| Ancillary and post-trial care | 30 | A compensation of 80 euros for cortical excitability ancillatory assessment is provided. In case of unexpected harm an insurance is established (SHAM company). |
| Dissemination policy | 31a | Trial results will be published in an international peer-reviewed journal in the field of psychiatry and neurostimulation. It could also be the subject of an oral communication in a congress. Patients enrolled can be informed of the conclusions of the study. |
|  | 31b | NA |
|  | 31c | NA |
| Appendices |  |  |
| Informed consent materials | 32 | Consent form has been validated by the ethical committee |
| Biological specimens | 33 | NA |

*It is strongly recommended that this checklist be read in conjunction with the SPIRIT 2013 Explanation & Elaboration for important clarification on the items. Amendments to the protocol should be tracked and dated. The SPIRIT checklist is copyrighted by the SPIRIT Group under the Creative Commons “[Attribution-NonCommercial-NoDerivs 3.0 Unported](http://www.creativecommons.org/licenses/by-nc-nd/3.0/)” license.
